# Role of G Protein-Coupled Receptors in Microglial Activation: Implication in Parkinson’s Disease

**DOI:** 10.3389/fnagi.2021.768156

**Published:** 2021-11-16

**Authors:** Chao Gu, Yajing Chen, Yan Chen, Chun-Feng Liu, Zengyan Zhu, Mei Wang

**Affiliations:** ^1^Department of Pharmacy, Children’s Hospital of Soochow University, Suzhou, China; ^2^Department of Child and Adolescent Healthcare, Children’s Hospital of Soochow University, Suzhou, China; ^3^Department of Neurology, Suzhou Clinical Research Center of Neurological Disease, The Second Affiliated Hospital of Soochow University, Suzhou, China

**Keywords:** G protein-coupled receptor (GPCR), microglial activation, Parkinson’s disease, neuroinflammation, dopaminergic (DA) neuronal loss

## Abstract

Parkinson’s disease (PD) is one of the prevalent neurodegenerative diseases associated with preferential loss of dopaminergic (DA) neurons in the substantia nigra compacta (SNc) and accumulation of α-synuclein in DA neurons. Even though the precise pathogenesis of PD is not clear, a large number of studies have shown that microglia-mediated neuroinflammation plays a vital role in the process of PD development. G protein-coupled receptors (GPCRs) are widely expressed in microglia and several of them act as regulators of microglial activation upon corresponding ligands stimulations. Upon α-synuclein insults, microglia would become excessively activated through some innate immune receptors. Presently, as lack of ideal drugs for treating PD, certain GPCR which is highly expressed in microglia of PD brain and mediates neuroinflammation effectively could be a prospective source for PD therapeutic intervention. Here, six kinds of GPCRs and two types of innate immune receptors were introduced, containing adenosine receptors, purinergic receptors, metabotropic glutamate receptors, adrenergic receptors, cannabinoid receptors, and melatonin receptors and their roles in neuroinflammation; we highlighted the relationship between these six GPCRs and microglial activation in PD. Based on the existing findings, we tried to expound the implication of microglial GPCRs-regulated neuroinflammation to the pathophysiology of PD and their potential to become a new expectation for clinical therapeutics.

## Introduction

Microglia are the main resident macrophages in the central nervous system (CNS), which originate from the yolk sac and merge into CNS at the early stage development (McGeer et al., 1988; Colonna and Butovsky, 2017; Prinz et al., 2019). Under physiological conditions, microglia make great contributions to the CNS homeostasis; however, in face of inflammatory stimuli, microglia rapidly transform their status into activated (Colonna and Butovsky, 2017; Wolf et al., 2017; Li and Barres, 2018). The link between microglia and PD was first reported 33 years ago and they found a mass of reactive microglia in PD brain (McGeer et al., 1988). Whereafter, a number of studies indicated that microglia-regulated neuroinflammation plays vital roles in PD pathogenesis (Jankovic, 2008; De Virgilio et al., 2016). Reactive microglia could be simply classified into two categories, including M1 phenotype and M2 phenotype (Ransohoff, 2016; Tang and Le, 2016), although this classification is not precise. M1 phenotype makes microglia detrimental with exaggerated pro-inflammatory factors, while M2 phenotype is neuroprotective and produces anti-inflammatory mediators like Arg1, IL-10, and CD206 (Jha et al., 2016; Tang and Le, 2016). In PD brain, excessive pro-inflammatory like interleukin-1β (IL-1β) and tumor necrosis factor-α (TNF-α) lead to severe damage to DA neurons (Sawada et al., 2006). Damaged DA neurons in turn stimulate microglial activation, resulting in a positive feedback loop of microglial activation and DA neuronal death (Glass et al., 2010). Although it is still undetermined whether neuroinflammation is the cause of PD pathogenesis or a downstream response of neuronal damage, some studies reported that inhibiting microglial action or clear activated microglia is neuroprotective and could relieve some motor symptoms in PD animal models (Gu et al., 2018), suggesting targeting neuroinflammation might show new lights on the research of PD pathogenesis.

Mammalian β-adrenergic receptor, as the first G protein-coupled receptor (GPCR), was identified in 1986 (Dixon et al., 1986). Nearly 800 members GPCRs are found and become the largest superfamily of cell surface receptors in mammals (Fredriksson et al., 2003; Nieto Gutierrez and McDonald, 2018). Almost 90% of the GPCRs can be bound by a wide range of neurotransmitters and neuromodulators, like norepinephrine, dopamine, and serotonin in brain (Doze and Perez, 2012). It is well documented that some microglial innate immune receptors, including Toll-like receptors (TLRs) and NOD-like receptors (NLRs) regulate microglial activation through the recognition of their ligands (Lan et al., 2017; Salvi et al., 2017; Ifuku et al., 2020; Ma et al., 2020). Recently, it has been reported that microglial TLR4 could mediate neuron-released α-synuclein clearance through selective autophagy (Choi et al., 2020). Interestingly, in the PD animal model, α-synuclein accumulation in microglia has been demonstrated to induce its activation by binding with several microglial innate immune receptors, such as CXCR4 (Li Y. et al., 2019). Numbers of endogenous or exogenous stimuli and perturbation could act on GPCRs, changing a wide variety of cell signaling, affecting the pathogenesis of many neurodegenerative disorders, including PD (Guixa-Gonzalez et al., 2012; Gandía et al., 2013; Azam et al., 2020). Moreover, G protein-coupled receptor kinases (GRKs) can act as the regulators of dopamine receptor functions, further influencing PD development (Gurevich et al., 2016). Currently, GPCRs have already been the targets of 34% Food and Drug Administration (FDA)-approved drugs available and some GPCRs have potential for clinical treatment in neurodegenerative disorders (Lütjens and Rocher, 2017; Hauser et al., 2018). Whether GPCRs also play roles in the regulation of microglia activation is worth further study.

In this review, we attempted to assess the relationship between GPCRs and neuroinflammation and the mechanisms underlying this phenomenon, as well as its importance in PD. We would like to discuss the role of GPCRs in microglial activation and we will focus on adenosine receptors, purinergic receptors, metabotropic glutamate receptors, adrenergic receptors, cannabinoid receptors, and melatonin receptors. We intended to focus on the therapeutic perspective of GPCRs as emerging drug targets for the development of novel therapeutic agents to PD treatment.

## Role of Adenosine Receptors in Microglial Activation of Parkinson’s Disease

Adenosine, as a neurotransmitter, is widely expressed in various cell types of CNS, including glial cells and neurons (Liu et al., 2019). Adenosine exerts physiological effects through four subtypes of adenosine receptors: A_1_, A_2__A_, A_2__B_, and A_3_ receptors, which belong to the purinergic GPCR family (Peleli et al., 2017). It is well known that adenosine acts as a vital role in neuronal excitability and synaptic transmission (Boison and Aronica, 2015); however, the adenosine-mediated role in glial function remains unclear. The dysfunction of adenosine receptors is reported to be closely related to PD pathogenesis (Cunha, 2016). Caffeine, as an adenosine antagonist, is neuroprotective against PD (Brothers et al., 2010; Bagga et al., 2016). In the MPTP-induced mouse model of PD, adenosine deaminase inhibition remarkably ameliorated DA neuronal death and improved motor disabilities (Huang et al., 2019).

A_1_ receptors are highly expressed in the cortex and hippocampus (Soliman et al., 2018). Even though there is no significant change of A_1_ receptors in the early stages of PD detected by ^11^C-MPDX PET (Mishina et al., 2017), an obvious increase of A_1_ receptors in the cerebellum was detected in virus-infected encephalitis rat model (Paul et al., 2014), suggesting the possible role of A_1_ receptors in some inflammatory-mediated pathological conditions. A_1_ receptors activation in microglia could suppress glioblastoma growth acting (Synowitz et al., 2006). Stimulation of A_1_ receptors inhibits microglial morphological activation through suppressing ATP-induced Ca^2+^ influx (Luongo et al., 2014), indicating the possible beneficial role of A_1_ receptors in microglia-mediated neuroinflammation. One study showed that paeoniflorin inhibits neuroinflammation via activating microglial A_1_ receptors in the MPTP mouse model (Liu et al., 2006), implying the protective role of A_1_ receptors in PD. Unlike A_1_ receptors, more research based on the relationship between A_2__A_ receptors and microglial activation was reported. In MPTP-treated mice, there is a significant increase in A_2__A_ receptor expression both in the substantia nigra (SN) and in the striatum (Gyoneva et al., 2014). Moreover, In lipopolysaccharide (LPS)-stimulated primary microglia, increased A_2__A_ receptors expression was also detected (Orr et al., 2009). Also, A_2__A_ receptors inhibition could suppress microglia activation in different neurodegenerative disorders (Gyoneva et al., 2014; Madeira et al., 2016), while A_2__A_ receptor activation could counteract the anti-inflammatory effects mediated by dopamine and further strengthen neuroinflammation (Meng et al., 2019). The combination of adenosine A_1__A_ receptor agonist and adenosine A_2__A_ receptor antagonist has a stronger inhibitory effect on neuroinflammation; among them, A_2__A_ antagonists have been used in clinical evaluation for the treatment of PD (Marucci et al., 2021). Interestingly, CD73-derived adenosine was reported to regulate inflammation and neurodegeneration (Meng et al., 2019).

Even though there is no definitive research of A_2__B_ and A_3_ receptors in PD pathogenesis, in LPS-treated microglia, A_2__B_ receptors activation has been reported to aggravate pro-inflammatory factors production through phosphorylation of cAMP response element binding (CREB) and promoting the p38 mitogen-activated protein kinase (MAPK) pathway (Koscsó et al., 2012; Merighi et al., 2017). The role of A_3_ receptor in microglia has also been reported. Activation of the microglial A_3_ receptors by adenosine or Cl-IB-MECA, a selective adenosine A_3_ receptor agonist, is capable of inhibiting TNF-α production through suppressing Akt and NF-κB activation in BV-2 microglia cells, while MRS1523, a selective A_3_ receptor antagonist, reverses this neuroprotective effect (Hammarberg et al., 2003; Lee et al., 2006).

## Role of Purinergic Receptors in Microglial Activation of Parkinson’s Disease

Purinergic receptors are categorized into two kinds of receptor families, containing ATP-gated ion channels (P2X) and GPCRs (P2Y) (Burnstock, 2017). P2X receptors are further subdivided into seven subtypes: P2X1, P2X2, P2X3, P2X4, P2X5, P2X6, and P2X7 receptors. GPCRs (P2Y) receptors are further subdivided into eight subtypes, including five Gq-coupled receptors: P2Y1, P2Y2, P2Y4, P2Y6, and P2Y11 and three Gi-coupled receptors: P2Y12, P2Y13, and P2Y14 (Burnstock, 2017). It is well reported that purinergic signaling is involved in the pathophysiology of CNS, including PD (Puchałowicz et al., 2014; Tóth et al., 2019). A recent study showed that purinergic receptor modulation could reverse aberrant Ca^2+^ signaling, might appear novel therapeutic potential for HD and PD (Glaser et al., 2020). P2X7 receptors are preferentially expressed on microglia compared to that on astrocytes and oligodendrocytes (Illes, 2020). P2X7 receptors are the amplifier of CNS damage in neurodegenerative diseases (Illes, 2020). In LPS-primed microglia, extracellular ATP generated from damaged tissue and astrocytes could bind to P2X7 receptors, leading to the inflammasome assembly and the releases of IL-1β and IL-18, which further make neurons damage (Heneka et al., 2018). In addition, P2X7 receptors in DA neurons are responsible for α-synuclein-induced oxidative stress and mitophagy impairment (Wilkaniec et al., 2020), implying its damage exacerbation in PD. P2X7 receptor antagonist can inhibit neuroinflammation and may be a potential drug target for the treatment of movement disorders (Fonteles et al., 2020), such as PD. Besides P2X7, P2X4 in the P2X receptor family is reported to regulate microglial function (Suurväli et al., 2017). In the experimental autoimmune encephalomyelitis (EAE) model, P2X4 receptors signaling inhibition or P2X4 knockout increases pro-inflammatory gene expression in microglia (Zabala et al., 2018). Moreover, P2X4 receptor protein is increased in the SN of the PD rat model and P2X4 receptor activation is involved in DA neuronal autophagy inhibition (Zhang X. et al., 2021), indicating that P2X4 receptor might serve as a potential way for the treatment of PD.

P2Y receptors, as G-coupled receptors are also well reported to tend to regulate microglial function (Von Kügelgen and Hoffmann, 2016). One study indicated that P2Y1 receptors might be the modulator of microglia–astrocyte interaction. It showed that microglia-derived cytokines could act on astrocytes, resulting in the downregulation of P2Y1 receptor and exerting neuroprotective effects (Shinozaki et al., 2017). Exogenous ATP triggered P2Y1 receptors activation, resulting in Ca^2+^ release from intracellular stores, making microglia to be pro-inflammatory (Orellana et al., 2013). In addition, both P2Y1 and P2Y12 are involved in ADP-induced migration of microglia mediated by transforming growth factor (TGF-β) (De Simone et al., 2010). There are very few studies about P2Y(2/4) receptors in neuroinflammation. Both P2Y2 and P2Y4 receptors are linked to amyloid beta (Aβ)-induced self-uptake by microglial pinocytosis, showing their more important role in AD (Kim et al., 2012; Li et al., 2013).

It seems that P2Y6 receptors play a more significant role than other P2Y receptors in PD.

P2Y6 receptors were markedly upregulated both in LPS-induced microglia and in the peripheral blood mononuclear cells (PBMCs) of PD patients (Yang et al., 2017). UDP, as the ligand of P2Y6, could promote microglial activation via the ERK1/2 pathway (Yang et al., 2017). Blocking UDP/P2Y6 receptor signaling could reverse these PD pathological processes (Yang et al., 2017; Anwar et al., 2020). It is also reported that a combination of P2Y6 and P2X7 receptor antagonists can be more protective in the 6-OHDA-induced PD rat model (Oliveira-Giacomelli et al., 2019). Moreover, P2Y6 receptors contribute to MPTP-induced neuronal SHSY5Y cell death (Qian et al., 2018). Therefore, P2Y6 receptor might be a potential clinical biomarker of PD. The role of P2Y12 and P2Y13 mediating microglial function has also been reported (Haynes et al., 2006; Zeng et al., 2014; Tatsumi et al., 2015). During neuropathic pain, P2Y12 receptor-dependent GTP-RhoA/ROCK2 signaling pathway upregulate excitatory synaptic transmission and microglial activation (Yu et al., 2019). A very recent study indicated that IL-1β release is increased remarkably in the P2Y13 knockout microglia while release evoked by LPS and ATP was not affected (Kyrargyri et al., 2020).

## Role of Metabotropic Glutamate Receptors in Microglial Activation of Parkinson’s Disease

It has been well reported that there is abundant glutamate in the CNS. Glutamate is an excitatory neurotransmitter maintaining communication and balance between glial cells and neurons (Ribeiro et al., 2017). Glutamate receptors are categorized into two types, namely, metabotropic glutamate receptors (mGluRs) and ionotropic glutamate receptors. mGluRs are further divided into three subgroups: Group I including mGlu1 and mGlu5 receptors; Group II including mGlu2 and mGlu3 receptors; and Group III consists of mGlu4, mGlu6, mGlu7, and mGlu8 receptors on the basis of sequence homology (Perfilova and Tyurenkov, 2016). mGluRs have been reported to be the therapeutic targets in PD (Litim et al., 2017). Among these mGluRs, Group III mGluRs activation could suppress glutamate release from presynaptic terminals of microglia, preventing neurons from excitotoxicity (Williams and Dexter, 2014). mGlu5 receptor seems to be closely linked to neuroinflammation. mGlu5 receptor is a potential regulator of microglial function and was first found in glial cells in 1999 (Biber et al., 1999). One study reported that microvesicles (MVs) released from microglia BV2 cells contribute to the communication between microglia and neurons and this interaction was mediated by mGlu5 receptor, resulting in the increased rotenone-induced neurotoxicity (Beneventano et al., 2017). This finding is really interesting. In the inflammatory-induced PD mouse model, the traditional Chinese medicine triptolide inhibits microglial activation via increasing mGlu5 receptor expression (Huang et al., 2018). Moreover, in the recent study of this group, they found that mGlu5 agonists inhibit α-synuclein-mediated neuroinflammation by regulating the binding of mGlu5 to α-synuclein (Zhang Y.N. et al., 2021), further indicating the significant role of mGlu5 in PD. Importantly, in mGlu5 receptor knockout (mGluR5^–/–^) mice, the increasing number of both microglia and astrocytes was found (Carvalho et al., 2019), implying that mGlu5 is involved in neuroinflammation.

## Role of Adrenergic Receptors in Microglial Activation of Parkinson’s Disease

Adrenergic receptors are divided into two groups, including α and β adrenergic receptors, and further subdivided into several kinds of subtypes, containing α_1_, α_2_ (subtypes α_2__A_, α_2__B_, and α_2__C_), β_1_, β_2_, and β_3_ (Strosberg, 1993). These adrenergic receptors exert physiological effects mainly through their ligand binding named noradrenaline (Strosberg, 1993). Dysfunction of the locus coeruleus noradrenergic system has been reported to be closely related to PD progression (O’Neill and Harkin, 2018; Sommerauer et al., 2018). Noradrenergic impairment contributes to the high prevalence of the non-motor symptoms in PD (O’Neill and Harkin, 2018). There are a few studies on the relationship between α-adrenergic receptors and microglial function. One study reported that dexmedetomidine, as a selective α_2_-adrenoceptor agonist, suppresses LPS-induced release of pro-inflammatory cytokines from hippocampal microglia specifically through α_2_-adrenergic receptor activation (Yamanaka et al., 2017). In another research, dexmedetomidine could regulate microglial polarization induced by 6-OHDA. Upon the pretreatment of dexmedetomidine, the inhibitory effects of 6-OHDA on IL-4-mediated induction of the anti-inflammatory factors, like IL-10 and IL-13, were remarkably alleviated, while the release of pro-inflammatory factors, such as IL-6 and IL-1β, were inhibited (Zhang et al., 2017).

Rather than α-adrenergic receptors, β-adrenergic receptors, especially β_2_-adrenergic receptors seem to be more closely linked to neuroinflammation in PD pathogenesis. The relationship between microglia and β-adrenergic receptors was first reported in 1988 (Fujita et al., 1998). They found that a β_2_-selective agonist terbutaline but not a β_1_-selective agonist restrains the microglial proliferation via enhancing intracellular cAMP level (Fujita et al., 1998). A number of studies reported that β_2_-adrenergic receptors inhibit microglia-mediated neuroinflammation via multiple signaling pathways to protect DA neurons from damage (Qian et al., 2009, 2011; Peterson et al., 2014; O’Neill et al., 2020). One of the studies showed that transformation from an M1- to M2-like phenotype in LPS-activated microglia by β_2_-adrenergic receptors agonists involves activation of the classical cAMP/PKA/CREB as well as the PI3K and p38 MAPK signaling pathways (Sharma et al., 2019). Another study reported that low doses of salmeterol inhibit microglial activation through a β_2_AR/β-arrestin2-dependent but cAMP/protein kinase A-independent pathway (Qian et al., 2011). A recent study indicated that β_2_-adrenergic receptors activation using different agonists dramatically alleviates the progression of dopaminergic neurodegeneration via inhibiting microglial activation in the LPS-challenged inflammatory PD mouse model (O’Neill et al., 2020).

## Role of Cannabinoid Receptors in Microglial Activation of Parkinson’s Disease

Cannabinoid (CB) receptors as classical G-protein coupled receptors are widely expressed throughout CNS, as well as certain parts of the body. Cannabinoid receptors mainly contain two subtypes, namely, CB1 and CB2 receptors (Zou and Kumar, 2018). A recent study showed that GPR55, an orphan receptor that can be activated by many classical cannabinoids, has been added as a third cannabinoid receptor (Moriconi et al., 2010). It is well documented that endocannabinoid as the endogenous ligand of cannabinoid receptors mediates large numbers of physiological effects through ligand-receptor binding (Zou and Kumar, 2018). Both Gi and Gs proteins were regulated by cannabinoid receptors activation (Howlett et al., 1986; Glass and Felder, 1997). A mass of studies regard cannabinoid receptors as the potential therapeutic target for PD (Baul et al., 2019; Cristino et al., 2020). CB receptors are equipped to play protective roles in different cellular stress in PD. Here, we focused on the roles of microglial CB1 and CB2 receptors, as well as GPR55 in neuroinflammation.

In MPTP-induced PD mouse model, pretreatment with non-selective CB receptors agonist WIN55212-2 or HU210 inhibited NADPH oxidase reactive oxygen species (ROS) production and reduced pro-inflammatory cytokines production from activated microglia, leading to increased DA neurons survival in the SN and improved the mouse motor function (Chung et al., 2011). The anti-inflammatory and neuroprotective effects were reversed upon treatment with CB1 receptor selective antagonist AM251 or SR14716A, confirming the importance of the CB1 receptor (Chung et al., 2011). It has been reported that the treatment of selective CB1 receptor antagonist SR141716A significantly increases pro-inflammatory factors (TNF-α, IL-1β, and IL-6) and chemokines (MCP-1 and CX3CL1) production in BV2 microglial cells through upregulating TLR4 and NF-κB/p65 expressions, further accelerating the clinical onset and development of EAE (Lou et al., 2018). However, one study showed that the anti-inflammatory effects of CB1 receptor might not be such important. In CB1 receptor knockout (*Cnr1*^–/–^) mice, less noradrenergic neurons in the locus coeruleus were found compared to their age-matched wild-type controls. Nevertheless, there was no enhanced pro-inflammatory profile in *Cnr1*^–/–^ mice even the density of microglia was increased (Gargano et al., 2020). Very interestingly, CB1 receptor was found to be localized on neuronal mitochondria, the G protein-coupled mitochondrial CB1 (mtCB1) receptors modulate memory processes through regulating mitochondrial energy metabolism (Hebert-Chatelain et al., 2016). This effect was mediated by mtCB1/mtGαi/PKA-dependent phosphorylation of complex I subunit NDUFS2 (Hebert-Chatelain et al., 2016). In their recent research, mtCB1 was also found in astrocytes, regulating glucose metabolism via phosphorylation of the mitochondrial complex I subunit NDUFS4 (Jimenez-Blasco et al., 2020). These two studies are indeed striking, as regulation of mitochondrial energy metabolism on microglial activation is currently a research hotspot. Whether microglial mtCB1 exists, if exists, will microglial mtCB1 modulate microglial activation through regulating mitochondrial energy metabolism mediated by certain molecules? This is really worthy of further study.

More research about CB2 receptors has been reported than CB1 receptors on microglia. One study reported that CB1 receptor gene expression was unchanged, while CB2Ar (A isoform, CB2Ar) gene expression was significantly increased (fourfold) in the SN of patients with PD (Navarrete et al., 2018). Another study also found the upregulation of CB2 receptors in microglia of SN in postmortem tissues of PD patients (Gómez-Gálvez et al., 2016). Moreover, in the rotenone or 6-OHDA-induced PD rat model, obvious upregulation of CB2 receptors expression was detected (Concannon et al., 2015, 2016). Interestingly, in the inflammatory rat PD model induced by Poly (I:C) or LPS, a more remarkable CB2 receptors increase was found (Concannon et al., 2015, 2016), implying that CB2 receptors were more influenced by inflammatory stimulation. These studies indicated targeting the CB2 receptor might represent a viable way for neuroinflammation modification in PD. CB2 receptor activation has been reported to inhibit neurotoxin-mediated neuroinflammation through regulating multiple molecules, including NRF2, ERK1/2, cPLA2, and NF-κB (Ribeiro et al., 2013; Galán-Ganga et al., 2020). Importantly, a much more intense deterioration of tyrosine hydroxylase (TH)-containing nigral neurons in CB2 receptor-deficient mice compared to wild-type animals (Gómez-Gálvez et al., 2016), indicating a potential neuroprotective role for CB2 receptor. The role of microglial GPR55 has also been reported recently. They found that KIT77, as an inverse agonist on GPR55 independent of the endocannabinoid system, significantly inhibited the release of PGE2 in primary microglia partially relying on the reduction of protein synthesis of mPGES-1 and COX-2 (Saliba et al., 2018). Recently, as the crystal structure of CB1 and CB2 receptors was analyzed (Krishna Kumar et al., 2019; Li X. et al., 2019), it becomes the guiding light for further research on CB receptors and really contributes to the drug discovery targeting CB receptors in PD.

## Role of Melatonin Receptors in Microglial Activation of Parkinson’s Disease

Melatonin is an amine hormone secreted mainly through the pineal gland in mammals (Hardeland et al., 2011). Circadian rhythm modulation and anti-oxidation effects have been thought to be the central physiological functions of melatonin (Reiter et al., 2000; Sanchez et al., 2015). G-protein coupled receptors (GPCR), namely, MT1 (encoded by *MTNR1A*) and MT2 (*MTNR1B*) regulate the majority of the biological functions of melatonin (Zlotos et al., 2014). Both MT1 and MT2 receptors are widely expressed in brain and play important roles in neurodegenerative diseases (Liu et al., 2016). It has been reported that MT1 receptors are reduced in AD patients (Wu et al., 2007). Silence of *MTNR1A* increases the amyloidogenic processing of amyloid precursor protein (APP) and exacerbates mutant huntingtin-mediated toxicity (Wang et al., 2011; Sulkava et al., 2018), indicating that MT1 plays a protective role in neurons. MT2 receptor is also closed related to AD pathogenesis. There was impaired hippocampal MT2 receptor signaling and MT2 activation ameliorated dendritic abnormalities through the cAMP-C/EBPα/miR-125b/GluN2A signaling pathway in AD (Tang et al., 2019). However, the role of MT receptors in PD pathogenesis.

It is well known that the relationship between melatonin and PD is mainly dependent on the anti-inflammatory and antioxidant effects. The majority of these neuroprotective effects on DA neurons in PD is receptor-independent and relies on antioxidant properties of the melatonin drug structure (Reiter et al., 2000, 2016; Pandi-Perumal et al., 2013). The role of MT1 and MT2 receptor in regulating PD pathogenesis, especially neuroinflammation remains unclear. Importantly, one study reported that there is a significant reduction of both MT1 and MT2 receptors in amygdaloid nucleus and SN of PD patients (Adi et al., 2010), possibly implying the significance of MT receptor in PD progression. Sleep disorder caused by circadian rhythm dysfunction is regarded as one of the vital non-motor symptoms of PD and often occurs in the early stage of PD progression (Fifel, 2017). Some researchers agree that dysfunction of circadian rhythm might be involved in PD pathogenesis (Lauretti et al., 2017). Both MT1 and MT2 receptors play vital roles in the regulation of circadian rhythm (Pandi-Perumal et al., 2007; Comai and Gobbi, 2014). Dysfunction of MT1 or MT2 might have the potential to participate in PD progression. The role in microglial MT1 on neuroinflammation is poorly understood. Our latest study indicated that microglial MT1 regulates microglial activation through PDHA1-mediated enhancement of oxidative phosphorylation (Gu et al., 2021). Others also reported that non-selective agonists such as ramelteon or agomelatine exert powerful anti-inflammatory effects (Molteni et al., 2013; Wang et al., 2019). Agomelatine, as a novel antidepressant, inhibits LPS-induced upregulation of pro-inflammatory factors via suppressing NF-κB nuclear translocation (Molteni et al., 2013). Ramelteon, which is used for treating insomnia clinically, protects neuronal degeneration in traumatic brain injury (TBI) through promoting nuclear factor erythroid 2-related factor 2 (Nrf2) nuclear accumulation, leading to the increasing of downstream factors of NRF2, including SOD-1, heme oxygenase-1, and NQO1 (Wang et al., 2019). It is very interesting that MT1 receptor is also located on neuronal mitochondria, like CB1 receptor. They found that melatonin is also synthesized in the mitochondrial matrix and released by the organelle to activate the mitochondrial MT1 signal-transduction pathway, thus inhibiting stress-mediated cytochrome c release and caspase activation, protecting neurons from death (Suofu et al., 2017). As microglial MT1 is closely linked to mitochondrial metabolic programming, there is the possibility that microglial mitochondrial MT1 exists and regulates mitochondrial-mediated microglial activation.

## Role of Innate Immune Receptors in Microglial Activation of Parkinson’s Disease

Except for the GPCRs mentioned above, innate immune receptors, also named pattern recognition receptors (PRRs), were widely expressed in innate immune cells, including microglia (De Nardo, 2015; Rodríguez-Gómez et al., 2020). It is well known that innate immune receptors are critical for modulating inflammatory responses (Rodríguez-Gómez et al., 2020). In regard to neurodegenerative diseases, TLRs and NOD-like receptors (NLRs), as best-characterized PRRs, play vital roles in neuroinflammation (Block et al., 2007; Kigerl et al., 2014; Kumar, 2019). Upon the binding of engaging ligands, microglial TLRs are activated, further causing internalization and conformational changes, leading to the recruitment of the adaptor proteins MyD88 or TRIF (TIR-domain-containing adaptor-inducing interferon-β) (De Nardo, 2015). Some transcription factors, including nuclear factor κB (NF-κB) are activated by downstream signaling cascades, resulting in subsequent production of pro-inflammatory cytokines (De Nardo, 2015). The relationship between TLRs and neuroinflammation is well clarified. For the pathogenesis of PD, TLRs are closely associated with α-synuclein. Misfolded or fibrillar α-synuclein released from neurons could be bound by the microglial TLR1/2 heterodimer, triggering NF-κB-mediated TNFα production (Kim et al., 2013). Another study also showed that fibrillar α-synuclein, but not oligomeric α-synuclein could promote IL-18 upregulation via a TLR1/2-dependent manner (Gustot et al., 2015). Microglial TLR4 also plays a significant role in α-synuclein-induced microglial activation, as α-synuclein could be uptake through TLR4, leading to pro-inflammatory cytokine release, and reactive oxygen species (ROS) production (Fellner et al., 2013). Furthermore, in the MPTP mouse PD model, TLR4 deficiency was neuroprotective (Noelker et al., 2013), indicating a detrimental role for TLR4 in PD pathogenesis.

Besides TLRs, NLRs mediate the process of the inactive IL-1β and IL-18 into active forms through inflammasomes activation, triggering pro-inflammatory responses (Latz et al., 2013). The NLR protein 3 (NLRP3) is the best-characterized inflammasome in PD, as in NLRP3-deficient mice, it was protective from MPTP-induced loss of DA neurons with decreased MPTP-induced caspase-1 activation and IL-1β release (Yan et al., 2015). There is extensive evidence that fibrillary α-synuclein, ATP, highmobility group box protein 1 (HMGB1), and lysophosphatidylcholine (LPC) could act as the activator of NLRs (Davalos et al., 2005; Freeman et al., 2017). Upon fibrillar α-synuclein insults, increased NLRP3 mRNA and protein expression were detected (Gustot et al., 2015; Zhou et al., 2016). This study suggested the key role of NLRP3 in PD pathogenesis.

## Conclusion

**FIGURE 1:**
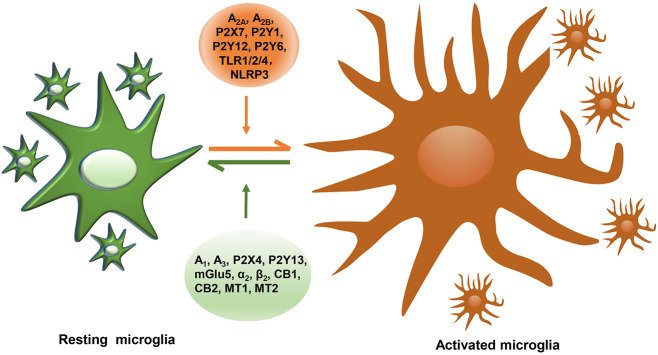
The summary of reported microglial GPCRs in microglial activation of PD. GPCRs expressed in microglia containing A_2__A_, A_2__B_, P2X7, P2Y1, P2Y12, P2Y6, TLR1/2/4, and NLRP3 act as detrimental roles, promoting microglial activation, damaging DA neurons, while microglial A_1_, A_3_, P2X4, P2Y13, mGlu5, α_2_, β_2_, CB1, CB2, MT1, and MT2 play protective roles in neuroinflammation.

In our present review, we mainly summarize six types of GPCRs and two types of innate immune receptors that are possibly related to disease progression of PD and their roles in mediating microglial action (Table 1). Some of these receptors, which consist of A_1_, A_3_, P2X4, P2Y13, Group III mGluRs, mGlu5, α_2_, β_2_, CB1, CB2, MT1, and MT2, act as beneficial roles and inhibit microglial activation, while A_2__A_, A_2__B_, P2X7, P2Y1, P2Y12, P2Y6, TLR1/2/4, and NLRP3 play unfavorable roles and are capable of inducing neuroinflammation (Figure 1). Drugs that target multiple receptors to inhibit microglial activation show considerable prospects in clinical therapeutic potential in PD. Very strikingly, CB1 and MT1 receptors were both found on mitochondrial outer membrane in neuron (Hebert-Chatelain et al., 2016; Suofu et al., 2017). Besides, CB1 receptor was also located on astrocytic mitochondria (Jimenez-Blasco et al., 2020). The possibility of microglial mitochondria is worthy to explore.

**TABLE 1:** Reported microglial GPCRs and their potential roles in PD.

**Microglial GPCRs**	**Sub-types**	**Ligands**	**Reported mechanisms**	**References**
Adenosine receptors	A_1_	Adenosine, paeoniflorin (agonist)	Promoting ATP-induced Ca^2+^ influx, neuroinflammation inhibition	Liu et al. (2006), Brothers et al. (2010), Luongo et al. (2014), Bagga et al. (2016)
	A_2A_	Preladenant SCH58261 Caffeine (antagonist)	Microglial activation inhibition, neuroprotective effects in MPTP model, increase of A_2A_ in MPTP or LPS-treated model	Orr et al. (2009), Brothers et al. (2010), Gyoneva et al. (2014), Bagga et al. (2016), Madeira et al. (2016)
	A_2B_	Adenosine, BAY60-6583 (agonist)	Promoting p-CREB and p-p38	Koscsó et al. (2012), Merighi et al. (2017)
	A_3_	Adenosine, Cl-IB-MECA (agonist)	Suppressing Akt and NF-κB activation	Hammarberg et al. (2003), Lee et al. (2006)
Purinergic receptors	P2Y1	ATP and ADP (agonist)	Increasing Ca^2+^ release from intracellular stores, promoting pro-inflammatory factors production	De Simone et al. (2010), Orellana et al. (2013)
	P2Y12	ADP (agonist)	TGF-β-induced microglial migration	De Simone et al. (2010)
	P2Y6	UDP (agonist)	P2Y6 upregulation in LPS-induced microglia, ERK1/2 activation, promoting MPTP-induced neuronal cell death	Yang et al. (2017), Qian et al. (2018), Anwar et al. (2020)
	P2Y12	P2Y12^–/–^mice	GTP-RhoA/ROCK2 signaling activation in P2Y12^–/–^ mice	Yu et al. (2019)
	P2Y13	P2Y13^–/–^microglia	IL-1β release is increased remarkably in P2Y13^–/–^ microglia	Kyrargyri et al. (2020)
Metabotropic glutamate receptors	mGlu5	CHPG (agonist)	Inhibiting α-synuclein-mediated neuroinflammation	Zhang Y.N. et al. (2021)
		Triptolide	Inhibiting microglial activation via increasing mGlu5 receptor expression	Huang et al. (2018)
		mGluR5^–/–^mice	Increasing number of both microglia and astrocytes	Carvalho et al. (2019)
Adrenergic receptors	α2	Dexmedetomidine (agonist)	Suppressing LPS-induced release of pro-inflammatory cytokines, regulating microglial polarization induced by 6-OHDA	Yamanaka et al. (2017), Zhang et al. (2017)
	β_2_	Terbutaline, Salmeterol, Clenbuterol, Formoterol (agonist)	Enhancing intracellular cAMP level, inhibit microglia-mediated neuroinflammation, activating classical cAMP/PKA/CREB as well as the PI3K and p38 MAPK signaling pathways, β2AR/β-arrestin2-dependent pathway, inhibiting microglial activation in LPS-challenged inflammatory PD mouse model	Fujita et al. (1998), Qian et al. (2011), Peterson et al. (2014), Sharma et al. (2019), O’Neill et al. (2020)
Cannabinoid receptors	CB1	Endocannabinoid, WIN55212-2, HU210 (agonist), SR141716A (antagonist)	Inhibiting NADPH and ROS, increasing pro-inflammatory factors (TNF-α, IL-1β, and IL-6) and chemokines (MCP-1 and CX3CL1)	Chung et al. (2011), Lou et al. (2018)
	CB2		Upregulation of microglial CB2 receptors in PD model	Concannon et al. (2015), Concannon et al. (2016), Gómez-Gálvez et al. (2016), Navarrete et al. (2018)
		WIN55212-2, Cannabinoids, CP55940 (agonist)	NRF2, ERK1/2, cPLA2, and NF-κB inhibition, neurotoxin-mediated neuroinflammation inhibition	Ribeiro et al. (2013), Galán-Ganga et al. (2020)
	GPR55	KIT77 (agonist)	Reduce protein synthesis of mPGES-1 and COX-2	Saliba et al. (2018)
Melatonin receptors	MT1	Ramelteon (agonist)	Enhancing PDHA1-mediated enhancement of oxidative phosphorylation	Gu et al. (2021)
	MT1/MT2	Agomelatine, Ramelteon, Melatonin, (agonist)	Suppressing NF-κB nuclear translocation, promoting Nrf2 nuclear accumulation	Molteni et al. (2013), Wang et al. (2019)

In our opinion, as mitochondrial metabolic dysfunction is not only related to DA neuronal death directly but also closely involved in microglial activation, moreover, both crystal structures of MT1 and CB1 were analyzed recently, we believe that agonists that bind to both MT1 and CB1 receptors have great potential for the development of clinical treatment of PD drugs.

## Author Contributions

CG and YJC wrote the manuscript. YC, ZZ, C-FL, and MW edited and revised the manuscript. All authors read and approved the final manuscript.

## Conflict of Interest

The authors declare that the research was conducted in the absence of any commercial or financial relationships that could be construed as a potential conflict of interest.

## Publisher’s Note

All claims expressed in this article are solely those of the authors and do not necessarily represent those of their affiliated organizations, or those of the publisher, the editors and the reviewers. Any product that may be evaluated in this article, or claim that may be made by its manufacturer, is not guaranteed or endorsed by the publisher.
